# Are Psychosocial Consequences of Obesity and Hyperandrogenism Present in Adolescent Girls with Polycystic Ovary Syndrome?

**DOI:** 10.1155/2018/3269618

**Published:** 2018-07-25

**Authors:** Agnieszka Zachurzok, Agnieszka Pasztak-Opilka, Elzbieta Forys-Dworniczak, Agnieszka Drosdzol-Cop, Aneta Gawlik, Ewa Malecka-Tendera

**Affiliations:** ^1^Department of Pediatrics and Pediatric Endocrinology, School of Medicine in Katowice, Medical University of Silesia, Katowice, Poland; ^2^Institute of Psychology, Faculty of Pedagogy and Psychology, University of Silesia, Katowice, Poland; ^3^Department of Woman's Health, School of Medicine in Katowice, Medical University of Silesia, Katowice, Poland

## Abstract

**Study Objective:**

The objective of this study was to evaluate whether body weight status and clinical hyperandrogenism may influence social competencies and psychological gender features in adolescent girls.

**Design and Participants:**

In 104 adolescent girls, psychological gender inventory (PGI) and social competencies questionnaire (SCQ) (assessing social abilities in three aspects: intimacy (I), social exposure (SE), and assertiveness (AS)) were performed. Subjects were divided into four subgroups: G1—24 nonobese girls without hyperandrogenism, G2—18 obese girls without hyperandrogenism, G3—30 nonobese hyperandrogenic girls, and G4—32 obese girls with hyperandrogenism.

**Results:**

There were no significant differences in all parts of SCQ and PGI between the study and control groups. The feminine woman type dominated in all groups; in G3 and G4, masculine woman type appeared more often than in G1 and G2 (13.3% and 12.5% versus 4.0% and 0.0%, resp.). In G4, positive relationship between BMI *z*-score and SCQ (*r* = 0.4, *p* = 0.03) was found. In G1, the relationship was opposite (*r* = −0.5, *p* = 0.03). Hirsutism correlated negatively with SCQ (*r* = −0.5, *p* = 0.02), I (*r* = −0.5, *p* = 0.02), and AS (*r* = −0.5, *p* = 0.02) only in G1; in other groups, this relationship was insignificant. In G4, higher testosterone level was associated with lower SCQ (*r* = −0.5, *p* = 0.008) and AS (*r* = −0.5, *p* = 0.003). In G2, testosterone concentration correlated positively with SCQ (*r* = 0.6, *p* = 0.01), SE (*r* = 0.5, *p* = 0.02), and AS (*r* = 0.6, *p* = 0.02).

**Conclusion:**

In adolescent girls, neither body weight nor clinical features of hyperandrogenism seem to be the source of evaluated disorders in psychological functioning.

## 1. Introduction

In today's culture, physical attractiveness is believed to be one of the main determinants of success. Having a high intelligence score, better social competencies, and good manner is attributed to good looks. Physical features characteristic of polycystic ovary syndrome (PCOS) that affects 5%–10% females in reproductive age, such as excessive body weight, hirsutism, and acne, are remote from the beauty standards [1, 2]. In the literature, psychological consequences of obesity in adolescents are wildly discussed especially stigmatization, lower quality of life, and depression [3–6]. However, the data are very often inconsistent, showing controversial results and indicating that factors other than obesity are responsible for psychosocial functioning of young women. In psychosocial functioning of girls and women with PCOS, psychological support of the family and the peer group plays an important role in self-image shaping.

In adolescent girls with PCOS, the lower self-evaluated quality of life and lower mood are present more often than in control subjects. Irregular menses, acne, hirsutism, obesity, and increased risk of infertility may lead to the deterioration of feminine identity [7]. As a consequence of obesity and hirsutism, lower sexual attractiveness, higher emotional distress, and higher depression score are frequently observed [4, 7, 8].

Social competencies, defined as the effective functioning abilities of the subject in social situations, are acquired during the process of social learning. Intelligence (social and emotional) and personality (extra- and introverted dispositions, reactivity, and fear) play a role in acquiring social competencies. Education, upbringing, and former therapies may also influence their shaping. Social competencies enable one to realize their own needs in a social acceptable manner. Self-esteem in adolescent girls is related to esteem of self-attractiveness and to establishment of interpersonal relationship. The acceptance of the surrounding society improves functioning, but the increase of criticism, especially in teenagers, deteriorates functioning, decreases self-esteem, and increases the level of anxiety and depression, leading to withdrawal from social relations. At the same time, negative image of somebody's body may impair the development of social competencies and lead to withdrawal from the intimate interrelationship [7, 9–17].

The adolescent years are also important for searching one's own identity, and gender identity plays a significant role in this process. Sandra Bem's gender schema theory [18] reflects the process of shaping of psychological features related to gender from the cultural perspective. Cognitive processes related to gender, which are shaped during the subject's development, make the classification to feminine and masculine type of behaviours possible, leading to self-concept creation. Thus, thru self-esteem, motivational processes are stimulated, which regulate behaviours according to cultural gender schema. There are four types of psychological gender identities: (1) sex-typed—individuals who process and integrate information that is in line with their gender, feminine woman/masculine man; (2) cross-sex-typed—individuals who process and integrate information that is in line with the opposite gender, feminine man/masculine woman; (3) androgynous—individuals having many characteristics of both male and female types; and (4) sexually undifferentiated—individuals with low scores in both male and female scales. Nowadays, quite common is the androgynic type of psychological gender, characterized by the presence of many feminine and many masculine attributes. It is associated with the minification of the role of gender differences in the social functioning [19–21].

The aim of the study was to determine social competencies and psychological gender features in lean and obese hyperandrogenic adolescent girls and compare them with lean and obese regularly menstruating and nonhirsute peers, as well as to evaluate the relationship between the psychological profile and clinical and hormonal components of PCOS.

## 2. Participants and Methods

Patients were recruited from the Department of Pediatrics and Pediatric Endocrinology of Medical University of Silesia from among the girls in the age of 14–18 years, who sought medical help for menstrual disorders and/or excessive body hair growth. Sixty-two consecutive adolescent girls with PCOS who agreed to participate were included in the study. The exclusion criteria were abnormal thyroid function, hyperprolactinaemia, congenital adrenal hyperplasia (based on basal 17-hydroxyprogesterone (17OHP) level < 3.0 ng/ml), usage of medications known to influence sex steroids in the last 3 months, and any known psychological disorders. Forty-two healthy, matched for age and BMI, regularly menstruating girls with no clinical signs of hirsutism served as controls. They were recruited from among the patients of Metabolic Clinic referred for the evaluation of lipid profile disturbances in whom hormonal imbalances were excluded. The refusal rate was 9.7% (*n* = 6) among the PCOS group and 16.7% (*n* = 7) among the healthy girls. The study was conducted according to Helsinki declaration and approved by the Ethics Committee of the Medical University of Silesia. Informed consent was obtained from each subject and parent or guardian. According to BMI and clinical symptoms of hyperandrogenism (menstrual disturbances and/or hirsutism), subjects were divided into 4 subgroups: group 1 (G1)—24 lean girls (BMI < 97th centile) without clinical features of hyperandrogenism, group 2 (G2)—18 obese girls (BMI > 97th centile) without clinical symptoms of androgen excess, group 3 (G3)—30 girls with normal body weight (BMI < 97th centile) but with clinical features of hyperandrogenism, and group 4 (G4)—32 obese girls (BMI > 97th centile) with menstrual disturbances and/or hirsutism.

### 2.1. Clinical and Hormonal Evaluation

We followed the methods of Zachurzok et al. [22]. In all participants, menstrual disturbances were evaluated and oligomenorrhea was defined as menstrual cycles longer than 45 days in the last six months (or less than 6 menstrual cycles during the last year). Weight was measured with seca scale with a precision of 100 g, and height measured with Harpenden stadiometer to 0.1 cm. BMI and BMI *z*-score were calculated. Hirsutism was evaluated and diagnosed if the modified Ferriman-Gallwey score was ≥8. In each girl, the transabdominal pelvic ultrasound examination was performed by the same observer (A.D-C.) with a 5 MHz convex transducer (Siemens Acuson Antares 5.0), and the volume and structure of the ovaries were evaluated. Ovaries were considered polycystic (polycystic ovary morphology (PCOM)) if 12 or more cysts (2–9 mm in diameter) were present at least in one ovary and if ovarian volume exceeded >12 ml. Basal plasma concentration of glucose, insulin, luteinizing hormone (LH), androstenedione (A), testosterone (T), 17OHP, and estradiol (E_2_) was measured. All the tests were performed during the follicular phase of menstrual cycle (3–7 days of cycle) or after 3 months from the last menstruation. Homeostatic model assessment of insulin resistance (HOMA-IR: fasting GLU (mmol/l) × fasting INS (mIU/l)/22.5) was calculated as the index of insulin resistance. PCOS was diagnosed according to the modified androgen excess and PCOS Society criteria [23]. A diagnosis of PCOS was made when all of the following criteria were present: menstrual disturbances (oligomenorrhea and amenorrhea), clinical or biochemical hyperandrogenism, and PCOM on ultrasound [24]. Serum levels of insulin, LH, T, DHEAS, and E_2_ were measured using chemiluminescent immunoassay by an Immulite 2000 analyzer (DPC, USA). 17OHP and A were measured by enzyme-linked immunosorbent assay (DRG Diagnostics GmbH, Germany).

### 2.2. Psychological Tests

In all participants, social competencies questionnaire (SCQ) [25] and psychological gender inventory (PGI) [26] were performed. The questionnaires were anonymous and independently answered during unlimited time, but the average time of completion was about 30 minutes.

The SCQ is a self-report questionnaire that was developed by Matczak [25] form the Psychological Test Laboratory of the Polish Psychological Association in Warsaw to assess social competencies in adolescents in 4-grade Likert's scale. It is designed to measure social competencies and consists of 90 sentences related to the participant's reaction to various situations, 60 diagnostics connected to social competencies, and 30 sentences irrelevant to social abilities. The questionnaire assesses the social competencies in three aspects: intimacy (I)—the ability to develop close interpersonal relationship and to share private information and feelings with another person, social exposure (SE)—connected to one's behaviour in formal situations and being an object of assessment of many people, and assertiveness (AS)—dealing with competencies for reaching one's own aims by influencing other people. The higher the social competencies, the higher the inner self-control in relation to success and coping with stressful situations. Higher social competency score is related also to high emotional intelligence. In adolescents, higher social competencies are related to higher involvement in social relations. Adolescent boys are usually characterized by higher assertiveness score, and adolescent girls are usually characterized by higher intimacy score [25].

The PGI is a self-report questionnaire that was developed by Kuczynska [26] from the Psychological Test Laboratory of the Polish Psychological Association in Warsaw, on the basis of Sandra Bem's gender schema theory to assess the sex-typical behaviours [18]. PGI is a Polish version of the Bem's sex role inventory. It consists of 35 adjectives: 15 feminine characteristics (cultural stereotype of femininity), 15 masculine characteristics (cultural stereotype of masculinity), and 5 features that are irrelevant to psychological gender (buffer items) assessed in the five-point scale.

Auxological data and hormonal and psychological test results were compared using the Statistica 8.0 PL. All values were expressed as mean (standard deviation) for normal distribution or median (interquartile range) for skewed distribution. Correlation analysis was performed using Pearson's correlation coefficient for normally distributed samples and Spearman's correlation coefficient for nonnormally distributed data. A comparison between groups was performed using Student's *t*-test for normally distributed data and Mann–Whitney *U* test for skewed distributions. Differences between four groups were assessed by one-way ANOVA or Kruskal-Wallis test, followed by the least significant difference (LSD) test for multiple comparisons when applicable. *p* value < 0.05 was considered statistically significant.

## 3. Results

The clinical and hormonal characteristics of the study participants are presented in Tables 1 and 2. All participants were Caucasian aged between 14.0 and 18.0 years (mean chronological age 16.4 ± 1.2 years). All girls from the study groups had at least one clinical symptom of PCOS (menstrual disturbances and/or hirsutism). As expected, hirsutism score, cycle duration, and LH, T, and A levels were significantly higher in girls with PCOS than in the control group (*p* < 0.05).

The results of SCQ and PGI are presented in Table 3. In G4, the general SCQ score was higher than in G1, but the difference did not reach statistical significance (*p* = 0.08). There were no significant differences in all parts of SCQ between the study and the control groups. Also in PGI, in both feminine and masculine gender scheme, the differences between the groups were statistically insignificant.

**Figure 1:**
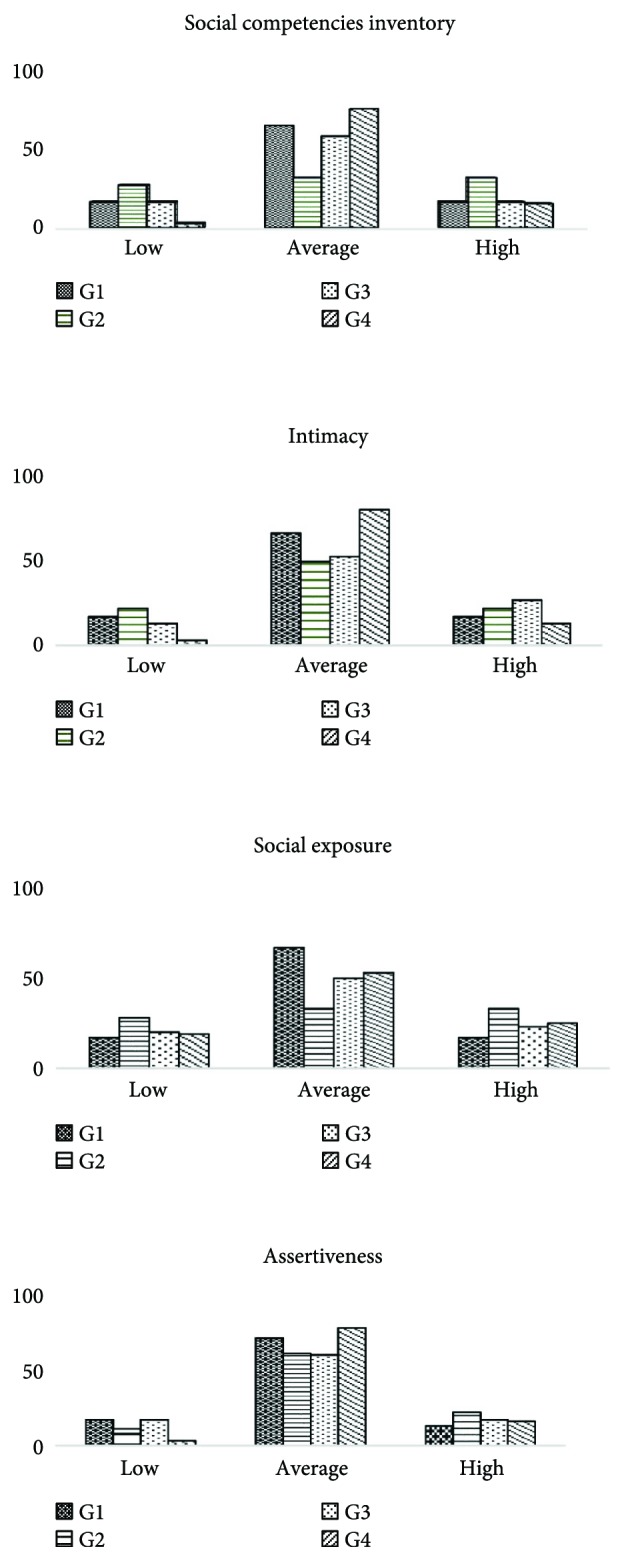
Distribution of low, average, and high results of social competencies inventory in study and control groups. G1: 24 lean girls (BMI < 97th centile) without clinical features of hyperandrogenism; G2: 18 obese girls (BMI > 97th centile) without clinical symptoms of androgen excess; G3: 30 girls with normal body weight (BMI < 97th centile) but with clinical features of hyperandrogenism; and G4: 32 obese girls (BMI > 97th centile) with menstrual disturbances and/or hirsutism.

In the control groups (G1 and G2), the most common were the average levels of social competencies (66.7% and 50.0% of subjects, resp.). Distribution of low, average, and high levels of competencies was normal (Figure 1). In the study groups (G3 and G4), the distribution of the social competencies was right skewed; however, the average level was also the most common in these groups (53.3% and 81.3% of subjects, resp.). In the SE scale, the distribution in all groups was similar, close to normal. However, in the I scale, in studied groups (G3 and G4), there was a very small number of subjects with low scores (9.4% and 6.7% of subjects, resp.) (right-skewed distribution). In G1 and G3, AS scores had normal distribution. However, in obese girls (G2 and G4), the distribution of this attribute was right skewed with very few girls with low scores (11.1% and 3.1% of subjects, resp.).

**Figure 2:**
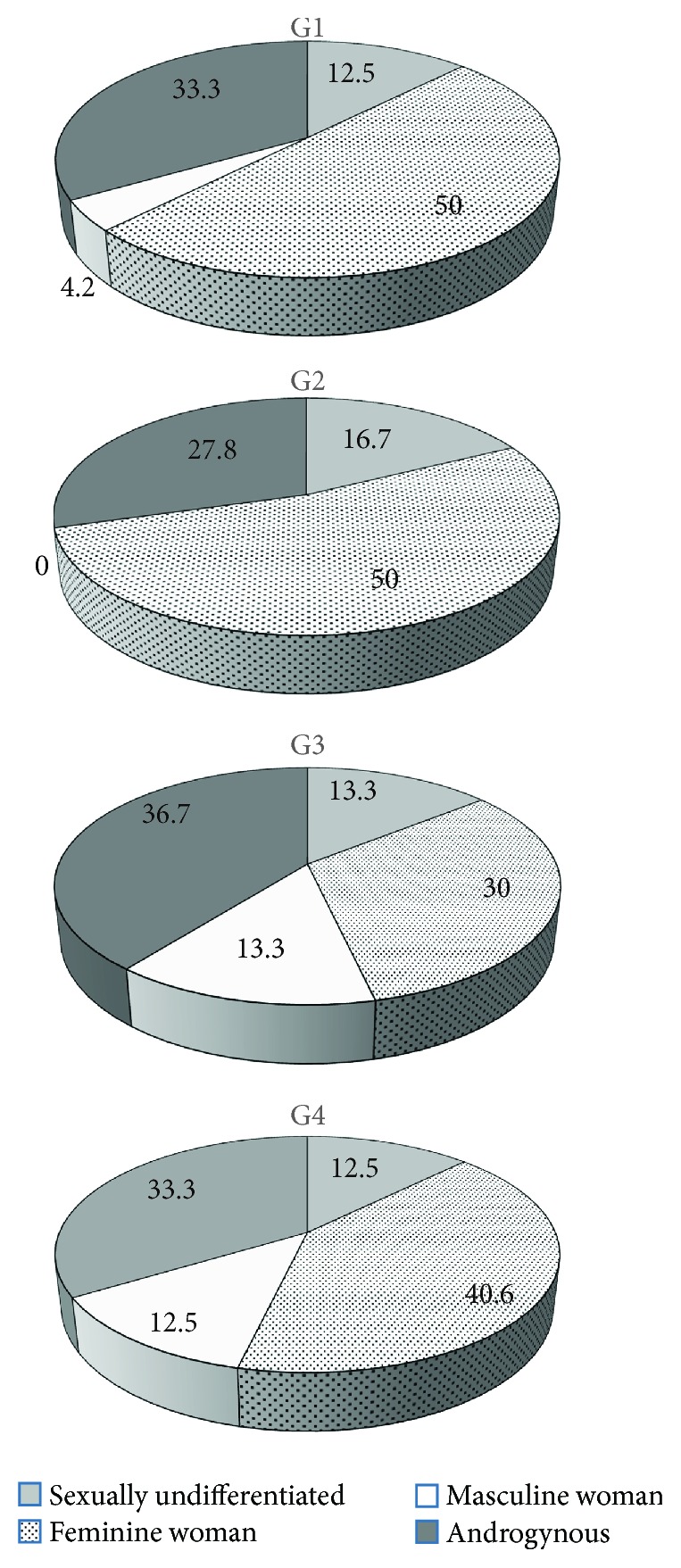
Distribution of types of psychological gender identities in study and control groups. G1: 24 lean girls (BMI < 97th centile) without clinical features of hyperandrogenism; G2: 18 obese girls (BMI > 97th centile) without clinical symptoms of androgen excess; G3: 30 girls with normal body weight (BMI < 97th centile) but with clinical features of hyperandrogenism; and G4: 32 obese girls (BMI > 97th centile) with menstrual disturbances and/or hirsutism.

The occurrence of the particular gender types was similar in all groups as presented in Figure 2. The feminine woman type dominated in all groups; however, in G3 and G4, the incidence of this type was slightly lower than in G1 and G2 (30.0% and 40.6% of subjects versus 50.0% and 50.0% of subjects, resp.). The reason was that the masculine woman type presented more often in G3 and G4 than in G1 and G2 (13.3% and 12.5% of subjects versus 4.0% and 0.0% of subjects, resp.).

**Figure 3:**
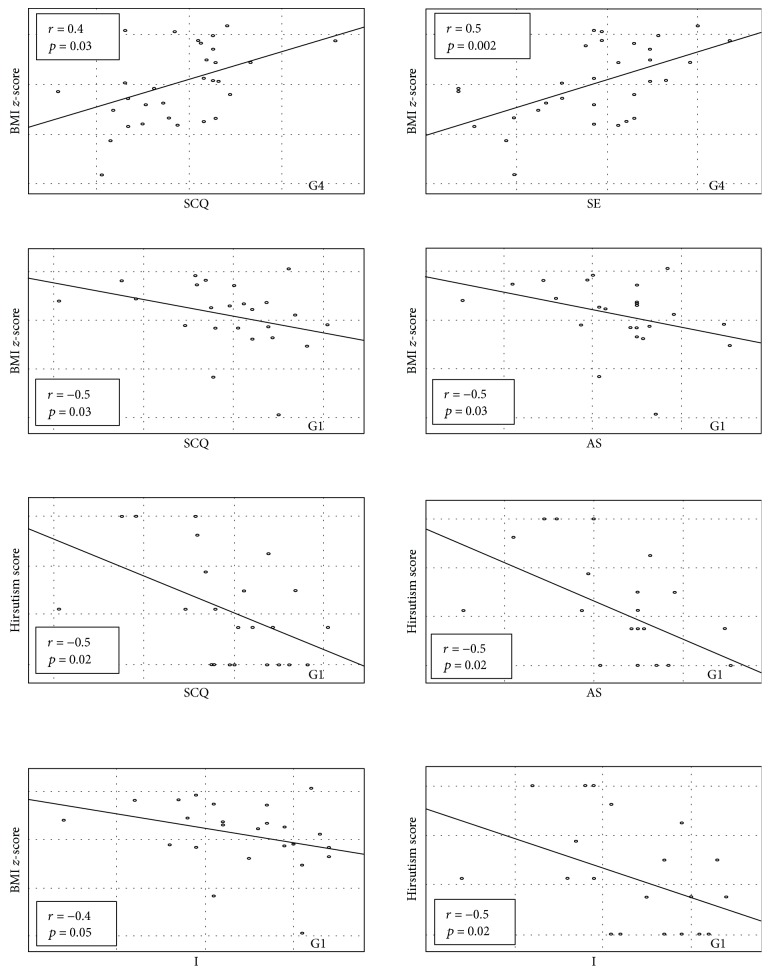
Correlation between BMI *z*-score and hirsutism score and social competencies questionnaire (SCQ) and its subscales (intimacy (I), social exposure (SE), and assertiveness (AS)) in G4 (32 obese girls (BMI > 97th centile) with menstrual disturbances and/or hirsutism) and G1 (24 lean girls (BMI < 97th centile) without clinical features of hyperandrogenism).

**Figure 4:**
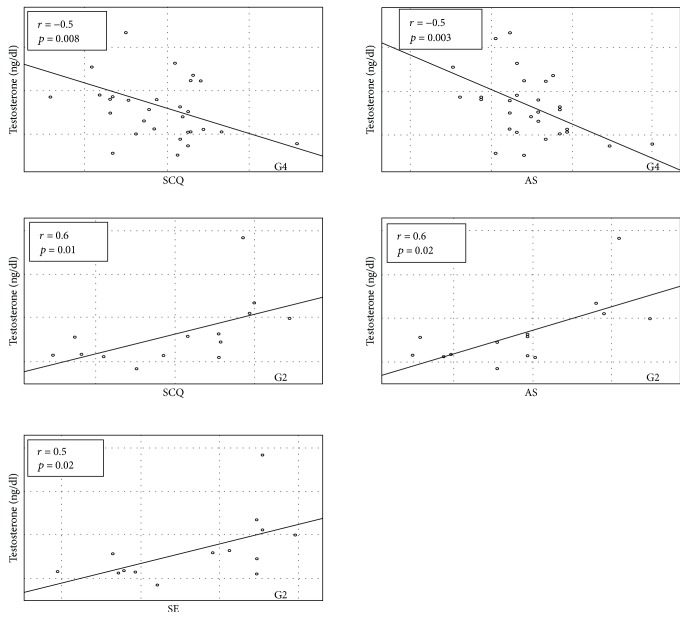
Correlation between testosterone level and social competencies questionnaire (SCQ) and its subscales (intimacy (I), social exposure (SE), assertiveness (AS)) in G4 (32 obese girls (BMI > 97th centile) with menstrual disturbances and/or hirsutism) and G2 (18 obese girls (BMI > 97th centile) without clinical symptoms of androgen excess).

In obese girls with androgen excess (G4), we found positive relationship between BMI *z*-score and general SCQ (*r* = 0.4, *p* = 0.03), especially SE score (*r* = 0.5, *p* = 0.002) (Figure 3). In G1, this relationship was reversed (*r* = −0.5, *p* = 0.03 for general SCQ, *r* = −0.4, *p* = 0.05 for I, and *r* = −0.5, *p* = 0.03) for AS scores. The hirsutism score correlated negatively with general SCQ, I, and AS scores only in G1—the group without significant hirsutism and with a hirsutism score in Ferriman-Gallwey of less than 7 (*r* = −0.5, *p* = 0.02; *r* = −0.5, *p* = 0.02; and *r* = −0.5, *p* = 0.02, resp.) (Figure 3). In other groups, also in those with significant hirsutism, the relationship between hirsutism score and social competencies was insignificant. However, in G4, higher T level was associated with lower social competencies in general SCQ as well as in the AS subscale (*r* = −0.5, *p* = 0.008 and *r* = −0.5, *p* = 0.003, resp.) (Figure 4). The relationship in obese girls without any clinical signs of androgen excess (G2) was opposite; T concentration correlated positively with general SCQ (*r* = 0.6, *p* = 0.01), SE (*r* = 0.5, *p* = 0.02), and AS score (*r* = 0.6, *p* = 0.02) (Figure 4). Moreover, in this group, T level was also related to higher scores in the masculine scale of psychological gender (*r* = 0.8, *p* = 0.001). In G2, also A and 17OHP concentrations were related to general SCQ (*r* = 0.6, *p* = 0.03 and *r* = 0.6, *p* = 0.04, resp.) and AS score (*r* = 0.7, *p* = 0.01 and *r* = 0.7, *p* = 0.02, resp.). Only in girls from control groups (G1 and G2), E_2_ level correlated with the feminine scale score (*r* = 0.5, *p* = 0.009) and T concentration correlated with the masculine scale score (*r* = 0.3, *p* = 0.05).

## 4. Discussion

In all the study groups, the most common psychological gender type was feminine women but about 1/3 of girls were of androgynous type, having many characteristics of both genders. In both study groups, G3 and G4, respectively, 13.3% and 12.5% of subjects presented with a masculine women type of psychological gender, and in both control groups, only one girl was defined as a masculine woman. This could indicate that elevated androgen level favours the masculine type of behaviours. Increased androgyny in psychological gender, besides biological determinants, could be caused by cultural and social gender stereotypes [19–21]. The cultural stereotypes of male and female gender concern three aspects of life: appearance, roles undertaken, and occupation. Tidiness, gentle voice, grace, elegance, and soft movements are considered to be feminine attributes in Polish culture, whereas being tall, strong, broad-shouldered, and vigorous is considered typical masculine attributes. However, in recent decades, these stereotypes have changed due to economic and political transformations, leading to the change in cultural roles assigned to male and female. Women are more often engaged in many life roles, coping with male together with female roles in everyday life. The present-day stereotype women's role not only includes raising up children and housekeeping but also includes working, which in the past was a typical male role [27]. Studies on androgynous women, who have features of both sexes, have shown that these women are more satisfied with life and more confident that they will be able to achieve their ambitions [19–21].

Manlove et al., in their pilot study, assumed that nowadays teenagers do not just engage in activities associated with their own gender but engage in many activities which were previously only associated with the other gender. This prepares them well for similar nonpolarising of roles in adulthood. The male stereotype of the gender role also refers to the girls with PCOS. They suggested that adolescent girls with PCOS tend to be “tomboys” during childhood, engaging in typical boy's games and activities rather than girl's [28]. This kind of behaviour tends to disappear with age as they adapt to cultural models of behaviour of their own gender; however, in doing so, they attain a lower sense of well-being. During their adolescent years, PCOS girls usually behave in a feminine manner. However, during adulthood, women with PCOS are more likely to have bisexual preference, which correlates in our results with the masculine women type found in adolescent girls [28].

The influence of obesity on psychological and social functioning of adolescent girls and adult women with PCOS is well documented by many studies [7–11, 14, 16, 17, 29]. The obesity leads to a decrease in quality of life and self-esteem, especially from a feminine point of view. Women, especially young ones, tend to judge themselves by the way they see themselves. Obese subjects with PCOS overreact to physical changes occurring in their body, which makes them distant from the ideal image presented in the media. This may lead to withdrawal from social interactions and increased depression score. However, our study revealed that in hyperandrogenemic obese girls, general SCQ scores tended to have the right-skewed distribution with a high number of subjects with high scores in general SCQ. In other groups, the distribution of the scores was normal. Moreover, in this group, the general SCQ score was higher than in nonobese girls without any clinical features of androgen excess, but the difference had only tendency to statistical significance. In G4, the increase of BMI *z*-score was related to higher social competencies, and in G1, this relationship was opposite—higher BMI *z*-score, but still within a normal range, was related to lower social competencies. Nonobese adolescent girls without hirsutism are especially sensitive to any body change and are characterized by the inclination to withdrawing from social relations when they notice subjective changes in their body image. These subjective changes, which are normally not confirmed by the environment, are the reason of seeking medical help. Obese girls, with BMI exceeding normal range, show opposite relation, and the excessive body weight does not affect their social functioning. This might be because of the social support that obese subjects get from medical and/or psychological institutions and family members, especially if the whole family suffers from obesity. It could also be due to subjective and positive self-assessment of their own body image [30–32]. This subjective body image, distant from the real picture of the body, could neutralize negative self-assessment. Long-lasting obesity could lead also to the feeling of “getting used” to the image of the obese body. The high rate of obesity in our society may make the obese child feel that he is not different from his peers.

Similar relationship may be present in the case of hirsutism as excessive body hair could lead to some disturbances in social and personal functioning. Jones et al. [9] found that a higher hirsutism score correlated with a lower quality of life and higher incidence of behaviours leading to getting rid of it (depilation, bleaching, etc.), especially in adolescent girls. Women with an increase sense of being unattractive tended to have a worse level of social functioning [9]. Surprisingly, we found that in G1, the hirsutism score, which was within a normal range, was associated with lower social competencies as well as lower assertiveness and intimacy. In obese girls without clinical signs of hyperandrogenism (G2), with serum concentration of androgens within the normal range in most subjects, the level of T, A, and 17OHP was positively related to social competencies and assertiveness. In G4, where T level was the highest, in many subjects exceeding the normal range, its concentration was related to the decrease of social competencies and assertiveness. It seems that when the androgen level remains within physiological limits, it favours social competencies and assertiveness. But when the androgen concentration exceeds normal range, the assertiveness sometimes converts to hostility and aggression, leading to a decrease in social functioning [33, 34]. Borghi et al. [35] showed that the level of anger and aggression is higher in women with PCOS than in the control group; however, the relationship between T level and anger was negative—the most elevated anger scores were associated with lower levels of androgen.

Hoyt and Kogan [36] observed that the highest satisfaction from intersexual contact was experienced not only by subjects of normal weight but also by obese and overweight subjects and underweight subjects, aspiring to have the ideal body image. Adolescent girls with normal body weight and without clinical hyperandrogenaemia seem to be overabsorbed with their body image. When they become aware of being different from the ideal body image, they tend to overreact emotionally, leading to a worsening social functioning and tendency to withdrawals from relationships. Frontini et al. [30] showed that in general, young women with a higher level of fear, hypersensitivity, and self-criticism presented a lower self-acceptance of their body and femininity and higher fear of interpersonal relationships. The self-acceptance in young women, despite the objective body image, enhances the interpersonal relationships, increases the feeling of self-control, facilitates the resolution of conflict, increases empathy in relationships, and increases life satisfaction. Higher self-assessment is connected with better functioning in intimate relationships. In women with poor acceptance of their own carnality, higher level of neurosis, fear, anger, and frustration was observed. The functioning in this group of women is worse in self-exposure because of the fear of defeat, problems with establishing social relationship, and loneliness in intimate contacts.

In our study, we found that the subjects from G1 were characterized by the least favourable psychological functioning profile. Paradoxically, these girls are the healthiest ones from a medical point of view with BMI within the normal range, without any clinical signs of hyperandrogenism, but at the same time, they are the ones who are the most focused on their body appearance. The exaggerated monitoring of their own body can lead to the increase in fear, shame, negative emotions, and depression [15].

In conclusion, in adolescent girls, neither the body weight nor the clinical features of hyperandrogenism seem to be a significant reason for evaluated disorders in psychological functioning but are rather most probably evoked by the adolescence period [37]. The reason for lack of relationship between PCOS and some aspects of psychological functioning could also be due to a short duration of clinical features or a short time of exposure to moderately elevated androgen concentration as well as other collateral factors influencing the social functioning and psychological gender features. The study limitation is a relatively small sample size, particularly in the healthy obese group. It might have been too small to demonstrate reliable estimates for several independent variables, and it could determine the inability to detect further subtle changes as well.

## Figures and Tables

**Table 1:** Clinical and hormonal characteristics of adolescent girls with polycystic ovary syndrome (PCOS) and control group of healthy girls.

	Girls with PCOS (*n* = 62)	Control group (*n* = 42)	*p*
Chronological age (years)	16.8 (15.8–17.3)	16.6 (15.0–17.0)	NS
Gynaecological age (months)	47.6 ± 19.7	48.8 ± 18.9	NS
Cycle duration (days)	57.5 (30.0–105.0)	28.0 (27.0–34.0)	<0.001
BMI *z*-score	1.4 ± 1.1	1.0 ± 1.3	NS
Ferriman-Gallwey score	19.0 (5.0–12.0)	2.0 (0.0–6.0)	<0.001
Mean volume of the ovaries (ml)	6.0 (4.8–8.0)	5.4 (3.8–7.6)	NS
LH (mIU/ml)	6.8 (3.3–10.5)	4.7 (3.1–6.7)	0.04
Testosterone (ng/dl)	57.5 (41.9–74.6)	45.4 (35.9–55.3)	0.005
Androstenedione (ng/ml)	4.3 ± 1.7	3.1 ± 1.4	0.002
DHEAS (*μ*g/dl)	287.7 ± 116.3	276.0 ± 90.8	NS
17OH progesterone (ng/ml)	1.6 (1.2–2.5)	1.6 (1.1–2.1)	NS

Note: values are mean ± standard deviation or median (interquartile range).

**Table 2:** Clinical and hormonal characteristics of adolescent girls with polycystic ovary syndrome (PCOS) (G3 and G4) and control groups of healthy girls (G1 and G2).

	G1 (*n* = 24)	G2 (*n* = 18)	G3 (*n* = 30)	G4 (*n* = 32)
Chronological age (years)	16.8 (15.8–17.3)	16.5 (14.3–17.0)	17.1 (16.2–17.5)	16.7 (15.6–17.1)
Gynaecological age (months)	45.0 (38.5–56.5)	58.5 (29.5–73.5)	50.0 (44.0–60.0)	47.0 (31.0–64.0)
Cycle duration (days)	28.0 (26.0–37.5)	28.5 (28.0–33.0)	60.0 (30.5–78.0)^1,2^	45.0 (30.0–180.0)^3,4^
BMI *z*-score	0.1 ± 0.9	2.2 ± 0.5^5^	0.6 ± 0.8^6^	2.3 ± 0.6^7,8^
Ferriman-Gallwey score	2.0 (0.0–1.0)	3.0 (0.0–6.5)	8.0 (3.0–11.0)^9,10^	10.0 (7.0–12.5)^11,12^
Ferriman-Gallwey score ≥ 8 (number (%))	0 (0%)	0 (0%)	17 (57%)	24 (75%)
Menstrual disturbances (number (%))	2.0 (8.3%)	0 (0%)	20 (66.7%)	21 (65.6%)
Testosterone (ng/dl)	50.5 ± 23.8	48.1 ± 26.6	59.6 ± 22.5	63.6 ± 30.1^13^
Androstenedione (ng/ml)	3.6 ± 1.5	2.8 ± 1.3	4.4 ± 1.9^14^	4.1 ± 1.5^15^
DHEAS (*μ*g/dl)	297.6 ± 79.7	261.0 ± 96.9	289.8 ± 90.6	285.9 ± 136.3
17OH progesterone (ng/ml)	1.9 (1.4–2.1)	1.5 (1.1–2.2)	1.6 (1.3–2.7)	1.5 (1.2–2.2)
Glucose (mg/dl)	84.4 ± 7.8	86.7 ± 7.4	86.0 ± 6.6	86.3 ± 9.4
Insulin (*μ*IU/ml)	9.4 (5.8–12.7)	18.8 (13.4–23.4)^16,17^	8.7 (5.7–11.8)	16.3 (10.7–20.3)^18,19^
HOMA-IR	1.9 (1.1–2.7)	4.1 (2.6–4.8)^20,21^	1.8 (1.1–2.4)	3.6 (2.4–4.2)^22,23^

Note: values are mean ± standard deviation or median (interquartile range). G1: 24 lean girls (BMI < 97th centile) without clinical features of hyperandrogenism; G2: 18 obese girls (BMI > 97th centile) without clinical symptoms of androgen excess; G3: 30 girls with normal body weight (BMI < 97th centile) but with clinical features of hyperandrogenism; and G4: 32 obese girls (BMI > 97th centile) with menstrual disturbances and/or hirsutism. HOMA-IR: homeostatic model of insulin resistance. ^1^G1 versus G3: *p* = 0.005. ^2^G2 versus G3: *p* = 0.02. ^3^G1 versus G4: *p* = 0.002. ^4^G2 versus G4: *p* = 0.009. ^5^G1 versus G2: *p* < 0.001. ^6^G2 versus G3: *p* < 0.001. ^7^G1 versus G4: *p* < 0.001. ^8^G2 versus G4: *p* < 0.001. ^9^G1 versus G3: *p* = 0.008. ^10^G2 versus G3: *p* = 0.01. ^11^G1 versus G4: *p* < 0.001. ^12^G2 versus G4: *p* < 0.001. ^13^G1 versus G4: *p* = 0.04. ^14^G1 versus G3: *p* = 0.001. ^15^G1 versus G4: *p* = 0.006. ^16^G1 versus G2: *p* = 0.003. ^17^G2 versus G3: *p* < 0.001. ^18^G1 versus G4: *p* = 0.01. ^19^G3 versus G4: *p* < 0.001. ^20^G1 versus G2: *p* = 0.002. ^21^G2 versus G3: *p* < 0.001. ^22^G1 versus G4: *p* = 0.01. ^23^G3 versus G4: *p* = 0.001.

**Table 3:** Social competencies inventory (SCI) and psychological gender inventory (PGI) results in study and control groups.

	G1 (*n* = 24)	G2 (*n* = 18)	G3 (*n* = 30)	G4 (*n* = 32)
Social competencies inventory (scores)	173.6 ± 30.2	180.1 ± 31.2	184.3 ± 22.5	186.3 ± 19.1
Intimacy (scores)	44.1 ± 7.6	45.8 ± 5.9	46.7 ± 7.0	46.3 ± 5.2
Social exposure (scores)	51.6 ± 11.5	53.0 ± 12.4	54.9 ± 9.2	55.5 ± 8.5
Assertiveness (scores)	47.0 ± 9.7	50.2 ± 9.0	49.2 ± 8.7	50.6 ± 6.5
Psychological gender inventory				
Feminine gender scheme (scores)	56.8 ± 7.4	55.4 ± 8.1	56.9 ± 7.4	56.0 ± 7.2
Masculine gender scheme (scores)	47.4 ± 9.4	46.2 ± 9.5	50.1 ± 8.2	47.6 ± 6.0

Note: values are mean ± standard deviation for normal or median (interquartile range) for skewed distribution. G1: 24 lean girls (BMI < 97th centile) without clinical features of hyperandrogenism; G2: 18 obese girls (BMI > 97th centile) without clinical symptoms of androgen excess; G3: 30 girls with normal body weight (BMI < 97th centile) but with clinical features of hyperandrogenism; and G4: 32 obese girls (BMI > 97th centile) with menstrual disturbances and/or hirsutism.

## Data Availability

The data used to support the findings of this study are available from the corresponding author upon request.
